# A Dual-Mode Pressure and Temperature Sensor

**DOI:** 10.3390/mi15020179

**Published:** 2024-01-25

**Authors:** Jin Chai, Xin Wang, Xuan Li, Guirong Wu, Yunlong Zhao, Xueli Nan, Chenyang Xue, Libo Gao, Gaofeng Zheng

**Affiliations:** 1 Xiamen Zehuo Digital Technology Co., Ltd., Xiamen 361102, China; cj@zehuo.cc; 2 School of Automation and Software Engineering, Shanxi University, Taiyuan 030006, China; 3 The 54th Research Institute of China Electronics Technology Group Corporation, Shijiazhuang 050051, China; 4 Pen-Tung Sah Institute of Micro-Nano Science and Technology, Xiamen University, Xiamen 361102, China; zhaoyunlong@stu.xmu.edu.cn (Y.Z.);

**Keywords:** tactile sensing, pressure, temperature, electronic skin

## Abstract

The emerging field of flexible tactile sensing systems, equipped with multi-physical tactile sensing capabilities, holds vast potential across diverse domains such as medical monitoring, robotics, and human–computer interaction. In response to the prevailing challenges associated with the limited integration and sensitivity of flexible tactile sensors, this paper introduces a versatile tactile sensing system capable of concurrently monitoring temperature and pressure. The temperature sensor employs carbon nanotube/graphene conductive paste as its sensitive material, while the pressure sensor integrates an ionic gel containing boron nitride as its sensitive layer. Through the application of cost-effective screen printing technology, we have successfully manufactured a flexible dual-mode sensor with exceptional performance, featuring high sensitivity (804.27 ${\mathrm{kPa}}^{-1}$), a broad response range (50 kPa), rapid response time (17 ms), and relaxation time (34 ms), alongside exceptional durability over 5000 cycles. Furthermore, the resistance temperature coefficient of the sensor within the temperature range of 12.5 °C to 93.7 °C is −0.17% °C^−1^. The designed flexible dual-mode tactile sensing system enables the real-time detection of pressure and temperature information, presenting an innovative approach to electronic skin with multi-physical tactile sensing capabilities.

## 1. Introduction

In recent years, with the advancement of sensor technology, tactile sensor technology has gradually emerged to mimic the sensing function of human skin. Flexible tactile sensors for force sensing can be divided into several types, such as piezoresistive [1,2,3], capacitive [4,5,6], piezoelectric [7,8,9], and optical sensors [10,11], according to the different conversion mechanisms between force and electrical signals. Each of these tactile sensors has different characteristics, and actual application scenarios require a flexible selection based on their advantages and disadvantages. For example, piezoresistive tactile sensors, which convert external forces into resistance signals for tactile perception, have been widely researched and applied due to their simple structure and low cost. In addition, flexible tactile sensors are also given the function of sensing the external environment to meet the needs of various complex scenes in the external environment. For example, thermoelectric, resistive, and thermistor flexible tactile sensors with temperature-sensing functions have been widely used in health monitoring and medical care. The proliferation of various flexible tactile sensors has led to the development of various manufacturing processes, including but not limited to screen printing, laser etching, 3D printing, and electrohydrodynamic (EHD) methods. In recent years, inkjet electrohydrodynamics (EHD) [12] has been widely used in the biomedical field due to its low voltage, low cost, and high resolution. In a study by Dong et al. [13], the PEDOT:PSS/Graphene (Gr)/Single-Walled Carbon Nanotubes multicomponent solution was directly written on the flexible PDMS substrate using the near-field electrohydrodynamic direct writing method, a serpentine shaped pressure sensitive unit was prepared and encapsulated with the PDMS thin film, and the flexible pressure sensor was fabricated.

To achieve multi-dimensional perception in flexible tactile sensors that meet diverse needs, researchers primarily endow them with the ability to perceive different detection objects through two methods: multi-functionalization of a single sensor or integration of multiple sensors. However, flexible tactile sensors [14,15] still face the challenge of inadequate integration. Most tactile sensors only detect specific physical quantities, such as pressure and temperature. Donghwa Lee et al. [16] developed an ultra-sensitive flexible piezoresistive sensor based on sea urchin-shaped metal nanoparticles (SSNPs). The sensor exhibits a sensitivity of 2.46 kPa^−1^ and an optical transmittance of 84.8% (550 nm), making it suitable for detecting small human muscle movements, such as hand movements. Soft piezoelectric capacitive sensors have garnered attention in the tactile sensor field due to advantages such as simple assembly and low power consumption. Weihao Zheng et al. [17] reported a stretchable ion pressure sensor (SIPS) array with low near-end crosstalk for epidermal monitoring. Due to the electric double layer (EDL) capacitance effect between the strong aramid nanofiber (ANF)/MXene (Ti_3_C_2_T_X_) composite electrode and the ion membrane, SIPS demonstrates high sensitivity up to 521.69 ${\mathrm{kPa}}^{-1}$, a low detection limit of 0.22 Pa, and a fast response time (17 ms). This indicates its significant potential for physiological monitoring in various life scenarios. Zheng Cui et al. [18] developed a flexible temperature sensor based on Ag nanowire composite material, capable of maintaining stable output under more than 1000 cyclic tensile tests and tensile strains up to 100%. Simultaneous detection of information such as pressure and temperature remains a substantial challenge. Therefore, the development of a multifunctional flexible tactile sensing system with excellent sensing performance and simultaneous detection of pressure and temperature information is a key focus of future research in tactile sensing technology [19,20].

Achieving dual functionality through a single process while maintaining independence is a challenge in multimodal sensor manufacturing. In this study, the singular manufacturing technique of screen printing was utilized to create a flexible dual-mode tactile sensor system, demonstrating the capacity for concurrent detection of both pressure and temperature. An interdigitated flexible pressure sensor with high sensitivity and wide response range was designed and fabricated based on thermoplastic polyurethane/1-ethyl-3-methylimidazolium bis(trifluoromethylsulfonyl)imide/hexagonal boron nitride (TPU/EMIM/h-BN). The sensor has ultra-high sensitivity (804.27 ${\mathrm{kPa}}^{-1}$), wide response range (50 kPa), fast response time (17 ms) and relaxation time (34 ms), and excellent durability under 5000 cycles. Using carbon nanotube/graphene conductive paste as the raw material, a flexible temperature sensor with stable performance was fabricated using a screen printing process. It has a resistance temperature coefficient of −0.17% °C^−1^ in the range of 12.5 °C to 93.7 °C, and is largely unaffected by bending deformation and humidity. Through structural optimization, the integrated design of flexible dual-mode sensors was achieved, and the feasibility of its application in the fields of wearable devices and electronic skin was verified.

## 2. Materials and Methods

### 2.1. Materials and Characterization

Highly elastic TPU film (XJU150, Shanghai Xingxia Polymer Products Co., Ltd., Shanghai, China) was chosen as the substrate material for the flexible dual-mode sensor. Stretched conductive silver paste (Shanghai Ouyi Organic Optoelectronic Materials Co., Ltd., Shanghai, China) was selected as the electrode material for the pressure sensor. TPU particles (Ruixiang Polymer Materials Business Department, Zhangmutou, Dongguan City, China) doped with ionic liquid (EMIM, Aladdin, Shanghai, China) and h-BN (Ningbo Bohuas Nano Technology Co., Ltd., Ningbo, China) were chosen as the sensitive layer materials for the pressure sensor [21]. Among them, the particle size of h-BN is 1–2 μm with a purity of 99.9%, and the particle size of TPU is 2–3 mm with a concentration of 97% for the ionic liquid. DMF (N,N-dimethylformamide, Aladdin) was chosen as the organic solvent. Carbon nanotube NMP ink (Suzhou Hengqiu Technology Co., Ltd., Suzhou, China) doped with a single-layer graphene (Shenzhen Suiheng Technology Co., Ltd., Shenzhen, China) was selected as the ink for screen printing the temperature sensor. 

The mechanical–electrical properties of the pressure sensor were assessed using an LCR digital instrument (TH2840B, Changzhou Tonghui Electronics Co., Ltd., Changzhou, China) and a ZhiQu mechanical testing machine (ZQ-990B). The temperature-electrical properties of the temperature sensor were evaluated using a heating stage (ZNCL-DL, Zhengzhou Biochemical Instrument Co., Ltd., Zhengzhou, China), a surface temperature detector (DT 1311, China Shenzhen Lihua Instrument Tools Co., Ltd., Shenzhen, China), and a digital meter (TH2840B, TongHui, Changzhou, China).

### 2.2. Structural Design

**Figure 1:**
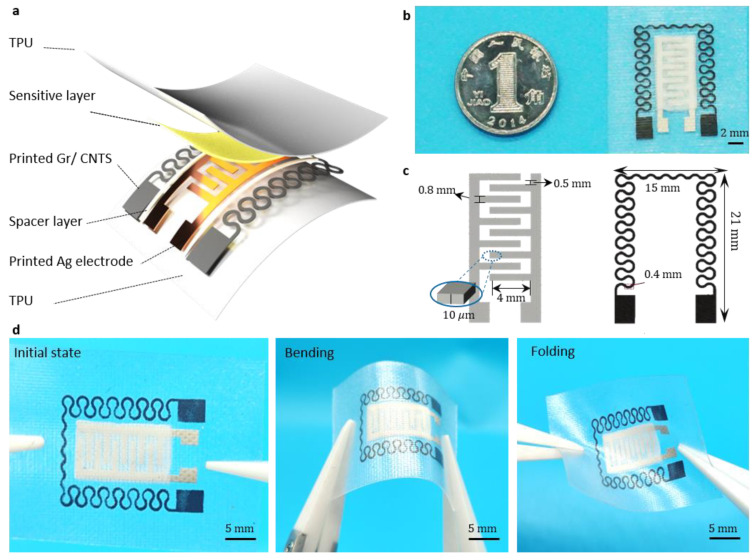
(**a**) Hierarchical structure and design principles of dual-mode sensors. (**b**) Physical picture of the dual-mode sensor. (**c**) Structural parameters of dual-mode sensors. (**d**) Physical image of a dual-mode sensor in its natural state, bending, and torsion.

The design of the flexible dual-mode sensor follows the configuration outlined in Figure 1a. The pressure sensor primarily consists of an interdigital electrode, a spacer layer, and a temperature sensor that is co-planarly integrated through a serpentine wire. The serpentine design of the temperature sensor is instrumental in minimizing the impact of strain on its resistance during bending or twisting. The actual dimensions of the sensor are shown in Figure 1b, while the structural parameters of the flexible dual-mode sensor are presented in Figure 1c. As illustrated in Figure 1d, the prepared dual-mode sensor exhibits ample flexibility to accommodate bending and twisting, ensuring adaptability to potential deformations during wear.

### 2.3. Preparation of Pressure Sensor

**Figure 2:**
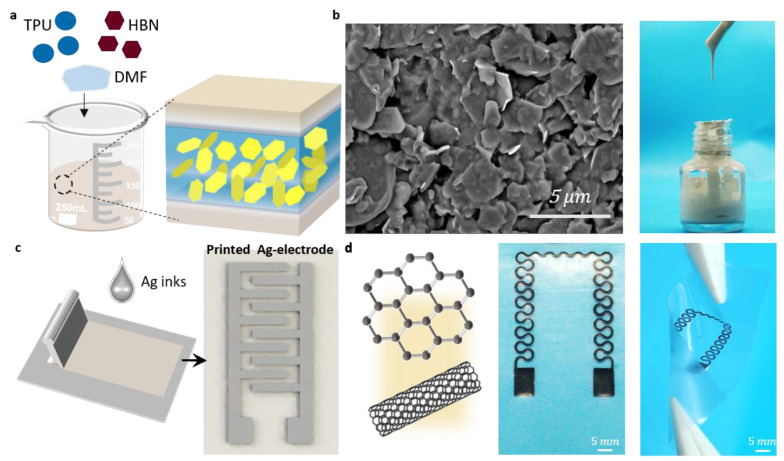
(**a**) The material composition. (**b**) SEM of the sensitive layer in the pressure sensor, and the physical picture of the ink material for printing the sensitive layer. (**c**) Cross-value electrodes in pressure sensors are mainly made by screen printing printable silver paste. (**d**) Material composition and physical picture of the temperature sensor.

The flexible pressure sensor consists of distinct layers: a sensitive layer, an electrode layer, and a base layer, arranged sequentially from top to bottom. The base layer is composed of a TPU film, while the electrode layer is formed by a screen-printed pattern of conductive silver paste. The sensitive layer comprises TPU/EMIM/h-BN ink obtained through the screen printing process. Specifically, 2 g of TPU particles were dissolved in 5 mL of DMF and stirred for 2 h at 80 °C and 1000 r/min. Then, 1 mL of EMIM solution was added, and stirring continued for an additional hour. Finally, 2 g of h-BN was introduced, and the mixture was heated and stirred for 2 h to produce the sensitive layer of ink (Figure 2a). The SEM and state diagram of the sensitive layer are depicted in Figure 2b.

Before printing the electrode layer, the substrate was cleaned using ethanol and deionized water (Figure 2c), and then screen printing took place. Likewise, the sensitive layer was printed on another clean substrate. The electrode layer and sensitive layer were placed on a heating table at 80 °C for 10 min to facilitate drying. Then, conductive silver adhesive and enameled wire were used to lead out the sensor electrodes, with double-sided adhesive serving as a spacer layer to complete the preparation of a flexible pressure sensor.

### 2.4. Preparation of Temperature Sensor

Through the incorporation of an appropriate quantity of graphene into a carbon nanotube slurry using NMP solvent, we fabricated a flexible temperature sensor with high sensitivity and rapid response speed, as shown in Figure 2d. Specifically, carbon nanotube slurry was thoroughly mixed and set aside. Graphene was accurately measured and added to the slurry. After 15 min of uniform stirring with a glass rod, a mixed ink with a graphene content of 2% by mass was prepared. The substrate was sequentially cleaned with ethanol and deionized water before being positioned on the printing table. The mixed conductive ink was applied to the screen printing screen for the temperature sensor, and printing was performed using a squeegee. The printed temperature sensor underwent drying on a heating table at 80 °C for 1 h. To finalize the preparation of the flexible temperature sensor, we used conductive silver glue and enameled wire to connect the sensor electrodes.

## 3. Results and Discussion

### 3.1. Performance of Pressure Sensor

**Figure 3:**
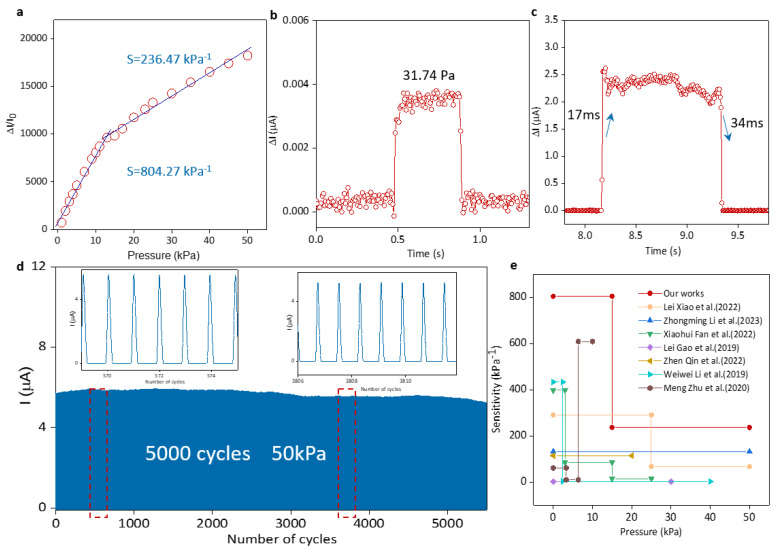
(**a**) Pressure sensor sensitivity and wide pressure application range. (**b**) The minimum detection limit of the pressure sensor. (**c**) Pressure sensor response time. (**d**) Attenuation of the pres-sure sensor after 5000 pressure cycles. (**e**) Sensitivity comparison of pressure sensors in this article and pressure sensors from other Refs. [1,30,31,32,33,34,35].

The sensor was fixed on the test platform of the mechanical testing machine using enameled wire to lead out the sensor electrode and LCR to test the sensor’s performance such as sensitivity [22,23], working range [24], response recovery time [25,26] and fatigue resistance [27] to characterize its performance in pressure detection ability. As one of the important indicators for evaluating the performance of flexible pressure sensors, sensitivity represents the ratio of the relative change in the sensor output signal to the pressure change, which is defined as $S=\left(\Delta I/I_{0}\right)/\Delta P$, where ΔI represents the flow through the change in current of the sensor, $I_{0}\;$ represents the initial current, and ΔP is the change in pressure. It can be seen from Figure 3a that as the applied pressure increases, the resistance of the sensor becomes smaller and the current increases. The maximum sensitivity can reach 804.27 ${\mathrm{kPa}}^{-1}$ in the range of 50 kPa. The ultra-high sensitivity proves its advantages in pressure sensing. Its minimum detection pressure is 31.74 Pa (Figure 3b), which proves its detection ability under weak signals.

Upon the application or removal of external pressure, the sensor’s output does not change immediately; instead, it stabilizes after a certain period. Response recovery time [28,29] indicates the sensor’s real-time response to instantaneous pressure. A shorter response recovery time indicates a stronger real-time response and the recovery capabilities of the sensor. Figure 3c illustrates that when an external force is rapidly applied and then removed, the sensor exhibits a response time of 17 ms and a recovery time of 34 ms, showcasing its excellent dynamic sensing capabilities for transient effects. Wearable electronic devices can aid in understanding pressure changes over time and responding promptly. Additionally, we conducted fatigue testing using a mechanical testing machine, subjecting the sensor to 5000 cycles of pressure at 50 kPa. Figure 3d demonstrates that the sensor’s output remained stable without significant decay during the testing process. The consistency between the sensor’s output before and after the test underscores its exceptional durability under extreme conditions, which reflects that the sensor can be reused. Simultaneously, this also indicates that the spacer layer within our sensor plays a crucial role in cyclic testing. Figure 3e illustrates the unique advantages of our prepared sensor compared with others, further confirming its excellent performance [1,30,31,32,33,34,35].

### 3.2. Performance of Temperature Sensor

**Figure 4:**
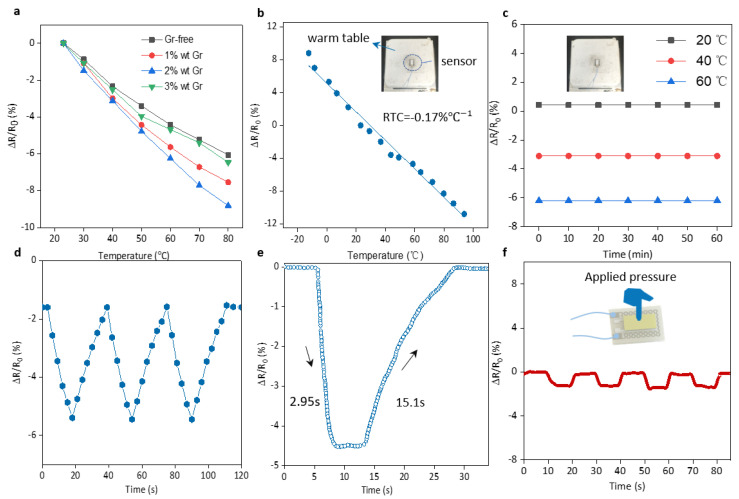
(**a**) Sensitivity comparison of temperature sensors doped with different concentrations of graphene in carbon nanotubes. (**b**) Temperature-resistance performance of temperature sensor doped with 2%wt graphene. (**c**) Temperature sensor undergoes a 1 h signal drift test at different temperatures. (**d**) Repeated high- and low-temperature experiments for temperature sensors. (**e**) Temperature sensor response time. (**f**) Effect of pressure on the temperature sensor.

The flexible temperature sensor adds an appropriate amount of graphene to the carbon nanotube slurry (NMP solvent) to control the sensor resistance. A sensor with moderate resistance is crucial for signal processing. Figure 4a shows the effect of doped graphene on the resistance of the temperature sensor. The results show that graphene doped with 2%wt has the best performance. Figure 4b shows the temperature-resistance performance of the temperature sensor doped with 2%wt graphene, showing excellent temperature-sensitive characteristics [36], and the resistance temperature coefficient (RTC) of the sensor under this ratio is −0.17% °C^−1^. Compared to some of the temperature sensors [37,38,39,40,41,42,43,44] already reported in Table 1, the temperature sensor developed in this study demonstrates superior performance. At the same time, we also conducted a temperature stability test and continuously measured the sensor current for 1 h at three constant temperatures of 20 °C, 40 °C, and 60 °C every 10 min. The sensor can maintain stable resistance at 20 °C, 40 °C, and 60 °C (Figure 4c). The small resistance change within 1 h proves its good stability. At the same time, the sensor was fixed on the surface of the heating stage, and repeated temperature rise and fall tests were performed in the temperature range of 30 °C to 60 °C. As the number of repetitions increased, the conductive network became more and more stable. As shown in Figure 4d, the resistance change rate of the sensor remained basically the same under the three temperature rising and cooling tests, proving its good repeatability and stability [45].

The assessment of response time stands as a pivotal metric in gauging the efficacy of the temperature sensor. In this context, the flexible temperature sensor was transitioned from an ambient room temperature state (23 °C) to an elevated condition on a heating table, registering a surface temperature of 50 °C. Subsequent signal detection and analytical scrutiny revealed a noteworthy response time of 2.95 s and a recovery time of 15.1 s for the flexible temperature sensor, as depicted in Figure 4e. This observation underscores the commendable responsiveness of the temperature sensor to fluctuations in external temperature.

To assess the stability of the flexible temperature sensor under various environmental conditions, we conducted tests to observe its resistance changes under pressure and bending deformation. The flexible temperature sensor is secured on the pressure test platform, subjecting it to a pressure of 50 kPa approximately every 10 s. Figure 4f illustrates that the test results indicate a resistance change rate of approximately −1.3% for the sensor under a pressure of 50 kPa. In comparison to the resistance change rate induced by temperature variations, the resistance change rate due to pressure is smaller, suggesting the sensor’s stability in real-world conditions.

### 3.3. Application of Dual-Mode Sensors

**Figure 5:**
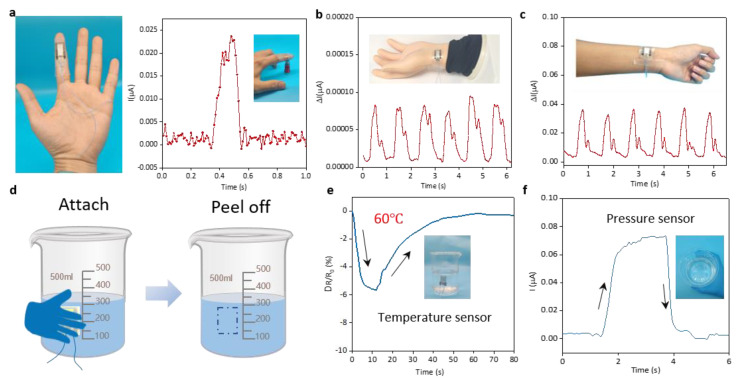
(**a**) Dual−mode sensors worn on human fingers realize tactile perception. (**b**) Dual−mode sensor worn on manipulator to measure pulse. (**c**) Dual−mode sensor worn on the human hand to measure pulse. (**d**) Schematic diagram of a dual-mode sensor attached to a 60 °C water cup. (**e**) Temperature signal changes in the dual-mode sensor before and after the dual-mode sensor is attached to a 60 °C water cup. (**f**) Changes in the pressure signal of the dual-mode sensor before and after the dual−mode sensor is attached to a 60 °C water cup.

As shown in Figure 5a, the flexible dual-mode sensor is worn on the finger and can fully realize tactile perception. In order to further verify the application potential of the sensor in human physiological information detection [46,47], and to realize the wearable function of the flexible sensor, the sensor was worn on the mechanical pulse arm and the radial artery of the human wrist. Figure 5b,c show that the sensor detects a stable pulse waveform, pulse wave contains rich physiological information, which is of great significance for the assessment of health status. It is worth noting that when the manipulator outputs a bionic pulse that is far lower than the human pulse lifting force (about 3% of the normal pulse amplitude), the sensor can still output the pulse stably, which fully demonstrates that the sensor can detect “sink” pulses. Excellent performance is conducive to full judgment and prediction of potential diseases.

In order to verify the sensor’s ability to detect temperature changes in the external environment, appropriate amounts of 60 °C hot water are added to the beaker, the sensor is worn on the finger, the beaker is picked up at room temperature, and it is put down after the sensor resistance stabilizes (Figure 5d). During the test, a temperature probe is used to measure the temperature of hot water in the beaker. Different beaker temperatures cause different changes in the resistance of the sensor. When picking up the beaker containing 60 °C hot water, the sensor resistance decreases from 14.8 kΩ to 14 kΩ, and the resistance change rate is about −5.6%. Through the real-time detection of the sensor current, the temperature of the outer wall surface of a glass containing 60 °C hot water was successfully detected (Figure 5e). When the sensor was attached/peeled off by hand on the cup wall, the flexible dual-mode sensor demonstrated excellent response capability under the combined influence of temperature and pressure (Figure 5f). This confirms the hybrid capability of the dual-mode sensor.

## 4. Conclusions

This paper presents the development of a flexible dual-mode sensing system for tactile perception, featuring information sensing capabilities for both pressure and temperature. Utilizing low-cost and easily implemented screen printing technology, we fabricated an interdigitated flexible pressure sensor based on TPU/EMIM/h-BN and a carbon nanotube/graphene-based flexible temperature sensor. The pressure sensor exhibits ultra-high sensitivity (804.27 ${\mathrm{kPa}}^{-1}$) with a wide response range (50 kPa). The temperature sensor demonstrates a resistance temperature coefficient of −0.17% °C^−1^ within the range of 12.5 °C to 93.7 °C, remaining nearly unaffected by bending deformation and humidity. In practical applications, the integrated dual-mode sensor excels in pulse detection and water cup temperature detection, showcasing its impressive tactile sensing capabilities as a wearable electronic device. Research indicates that enhancing tactile sensing can be achieved through multi-sensor fusion. Integrating sensors for humidity, slip, and vibration to broaden and integrate tactile sensing capabilities represents a promising direction for the future development of wearable electronic skins.

## Figures and Tables

**Table 1:** Some reported temperature sensors.

Material	Method	Measurement Range (°C)	RTC (%/°C)	Ref.
MWCNTs/GNP	Vacuum filtration	30–100	0.138	[37]
Graphene-PEDOT: PSS/PU	Inkjet printing	35–45	0.06	[38]
MWCNTs/PVDF	Printing/dip coating	20–120	0.13	[39]
Hybrid thread (PES-Steel microwire)	Embroidery	40–120	0.1	[40]
Polyvinyl chloride/carbon black (PVC/CB)	Screen printing	18–44	−0.148	[41]
AgNPs	Inkjet printing	−20–60	0.11	[42]
PZT/PDMS	Inkjet printing	25–120	0.0875	[43]
rGO/AgNPs	Inkjet printing	30–100	0.1	[44]
CNT/graphene	Screen printing	12.5–93.7	−0.17	Our work

Notes: Multi-walled carbon nanotubes (MWCNTs); graphene nanoplatelets (GNP); poly(3,4-ethylenedioxythiophene):poly (styrenesulfonate) (PEDOT: PSS); polyurethane (PU); polyvinylidene fluoride (PVDF); Ag nanoparticles (AgNPs); piezoelectric lead zirconate titanate/polydimethylsiloxane (PZT/PDMS); reduced graphene oxide/Ag Nanoparticles (rGO/AgNPs); Carbon nanotube (CNT).

## Data Availability

The data that support the findings of this study are available upon request from the corresponding author upon reasonable request.
